# Generalizability of Polygenic Risk Scores for Breast Cancer Among Women With European, African, and Latinx Ancestry

**DOI:** 10.1001/jamanetworkopen.2021.19084

**Published:** 2021-08-04

**Authors:** Cong Liu, Nur Zeinomar, Wendy K. Chung, Krzysztof Kiryluk, Ali G. Gharavi, George Hripcsak, Katherine D. Crew, Ning Shang, Atlas Khan, David Fasel, Teri A. Manolio, Gail P. Jarvik, Robb Rowley, Ann E. Justice, Alanna K. Rahm, Stephanie M. Fullerton, Jordan W. Smoller, Eric B. Larson, Paul K. Crane, Ozan Dikilitas, Georgia L. Wiesner, Alexander G. Bick, Mary Beth Terry, Chunhua Weng

**Affiliations:** 1 Department of Biomedical Informatics, Columbia University Irving Medical Center, New York, New York; 2 Department of Epidemiology, Columbia University Irving Medical Center, New York, New York; 3 Division of Medical Oncology, Rutgers Cancer Institute of New Jersey, Robert Wood Johnson Medical School, New Brunswick, New Jersey; 4 Department of Pediatrics, Columbia University Irving Medical Center, New York, New York; 5 Department of Medicine, Columbia University Irving Medical Center, New York, New York; 6 National Human Genome Research Institute, Bethesda, Maryland; 7 Department of Medicine, University of Washington, Seattle; 8 Department of Population Health Sciences, Geisinger, Danville, Pennsylvania; 9 Genomic Medicine Institute, Geisinger, Danville, Pennsylvania; 10 Department of Bioethics and Humanities, University of Washington, Seattle; 11 Psychiatric and Neurodevelopmental Genetics Unit, Center for Genomic Medicine, Massachusetts General Hospital, Boston, Massachusetts; 12 Kaiser Permanente Washington Health Research Institute, Seattle, Washington; 13 Department of Cardiovascular Medicine, Mayo Clinic, Rochester, Minnesota; 14 Department of Medicine, Division of Genetic Medicine, Vanderbilt University Medical Center, Nashville, Tennessee

## Abstract

**Importance:**

Multiple polygenic risk scores (PRSs) for breast cancer have been developed from large research consortia; however, their generalizability to diverse clinical settings is unknown.

**Objective:**

To examine the performance of previously developed breast cancer PRSs in a clinical setting for women of European, African, and Latinx ancestry.

**Design, Setting, and Participants:**

This cohort study using the Electronic Medical Records and Genomics (eMERGE) network data set included 39 591 women from 9 contributing medical centers in the US that had electronic medical records (EMR) linked to genotype data. Breast cancer cases and controls were identified through a validated EMR phenotyping algorithm.

**Main Outcomes and Measures:**

Multivariable logistic regression was used to assess the association between breast cancer risk and 7 previously developed PRSs, adjusting for age, study site, breast cancer family history, and first 3 ancestry informative principal components.

**Results:**

This study included 39 591 women: 33 594 with European, 3801 with African, and 2196 with Latinx ancestry. The mean (SD) age at breast cancer diagnosis was 60.7 (13.0), 58.8 (12.5), and 60.1 (13.0) years for women with European, African, and Latinx ancestry, respectively. PRSs derived from women with European ancestry were associated with breast cancer risk in women with European ancestry (highest odds ratio [OR] per 1-SD increase, 1.46; 95% CI, 1.41-1.51), women with Latinx ancestry (highest OR, 1.31; 95% CI, 1.09-1.58), and women with African ancestry (OR, 1.19; 95% CI, 1.05-1.35). For women with European ancestry, this association with breast cancer risk was largest in the extremes of the PRS distribution, with ORs ranging from 2.19 (95% CI, 1.84-2.53) to 2.48 (95% CI, 1.89-3.25) for the 3 different PRSs examined for those in the highest 1% of the PRS compared with those in the middle quantile. Among women with Latinx and African ancestries at the extremes of the PRS distribution, there were no statistically significant associations.

**Conclusions and Relevance:**

This cohort study found that PRS models derived from women with European ancestry for breast cancer risk generalized well for women with European, Latinx, and African ancestries across different clinical settings, although the effect sizes for women with African ancestry were smaller, likely because of differences in risk allele frequencies and linkage disequilibrium patterns. These results highlight the need to improve representation of diverse population groups, particularly women with African ancestry, in genomic research cohorts.

## Key Points

**Question:**

How do previously developed breast cancer polygenic risk scores (PRSs) perform in a clinical setting for women of different ancestries?

**Findings:**

In this multicenter cohort study linking electronic medical records to genotyping data that including 39 591 women, PRSs were significantly associated with breast cancer risk in women of all ancestries, although the effect sizes were smaller in women with African ancestry.

**Meaning:**

Previously developed PRS models for breast cancer risk performed well for women with European and Latinx ancestries in different clinical settings; these results suggest that larger studies are needed to develop and validate PRSs for women with African ancestry.

## Teaser

This cohort study of multiple US medical centers with electronic medical records linked to genotype data examines the association of the ancestry of populations used to model polygenic risk scores for breast cancer with their ability to estimate cancer risk for women with different racial/ethnic ancestries.

## Introduction

Polygenic risk scores (PRSs) have consistently shown the ability to stratify the risk of breast cancer among women with European ancestry,^1^ but their generalizability to other race/ethnic groups is more limited. For example, using large consortia of women with European ancestry, a PRS developed in the Breast Cancer Association Consortium (BCAC), reported approximately 2-fold and 4-fold increases in breast cancer risk for women in the top 10% and 1% of the PRS respectively; compared with women in the middle quantiles of risk (40% to 60%).^2^ This association has been replicated in validation studies using large cohorts of women with European ancestry.^3,4^

Understanding the performance of these PRSs in diverse populations is crucial as we move toward clinical implementation of the PRS. In order to incorporate PRSs into clinical practice, models will need to be integrated with other clinical covariates like family history in the electronical medical records (EMR).^5^ With few exceptions,^6^ studies have not yet evaluated the performance of breast cancer PRSs using clinical data extracted from the EMR.

The Electronic Medical Records and Genomics (eMERGE) network is a federated network of academic medical centers in the US and has compiled EMRs and genotype data for genomic research.^7^ By using the rich resources of the eMERGE network, including the extensive breast cancer phenotyping algorithm and a diverse population assembled across the network’s federated sites, this study aims to provide a systematic evaluation of the generalizability of previously developed breast cancer PRSs for women of European, African, and Latinx ancestry.

## Methods

### Study Participants

The participants involved in this cohort study were women enrolled through the eMERGE network from 9 contributing US medical centers with EMRs linked to genotype data. We identified breast cancer cases and controls through a validated phenotyping algorithm. We established ancestry by requiring the observed/self-reported ancestry to match the genetic ancestry inferred by principal component analysis-based k-means group, as previously described.^8^ Note that for Latinx women, we only used self-report because of the diversity of admixture genetic background. We did not include Asian, American Indian/Native American, Native Hawaiian/Pacific Islander, and other ancestry groups in this study given the corresponding small number of breast cancer cases.

This study was conducted in accordance with the principles of the Declaration of Helsinki.^9^ The institutional review board of each contributing institution approved the eMERGE study, and the Columbia University Health Sciences institutional review board approved this study because analysis was conducted using deidentified data. All participants provided written informed consent prior to study inclusion. A specific discussion of the ethical considerations across the eMERGE III study is described elsewhere.^10^ This study followed the Strengthening the Reporting of Observational Studies in Epidemiology (STROBE) reporting guideline.

### PRS Models

We examined the performance of 7 PRS models previously developed and tested in women with European, African, or Latinx ancestries (Table 1). We reconstructed each PRS based on included variants and corresponding effect sizes in the original publications and used PLINK version 1.9^15,16^ to calculate each PRS (more details in the eMethods in the Supplement). We included 3 PRS models developed in women with European ancestry (2 developed from BCAC data with small and large numbers of variants [BCAC-S and BCAC-L, respectively]^2^ and 1 from UKBiobank data [UKBB]^11^), which included 313, 3820, and 5218 variants, respectively. We also included 2 PRS models developed in or adapted to Latinx women (Women’s Health Initiative [WHI-LA], 71 variants^13^; and a model developed by Shieh et al^12^ including multiple cohorts of US Latina and Latin American women [LATINAS], 179 variants), as well as 2 PRS models developed in women with African ancestry (WHI cohort of women with African ancestry [WHI-AA],^13^ 75 variants; and a cohort from the African Diaspora study conducted by the Root consortium [ROOT],^14^ 34 variants) (Table 1). For women with European ancestry, we also evaluated PRSs developed for estrogen receptor (ER)-positive and ER-negative breast cancers.^2^

**Table 1.** Seven Polygenic Risk Score (PRS) Models Previously Developed for Women With European Ancestry or Optimized for Other Ancestries

PRS Models	No. of Variants^a^	Source	Validation, No. in cohort (No. of cases)^b^	Ancestry of Validation Cohort
BCAC-L	3820 (2532)	Mavaddat et al,^2^ (2019)	18323 (11 428)	European
BCAC-S	313 (209)			
UKBB	5218 (4192)	Khera et al,^11^ (2018)	157895 (6586)	European
LATINAS	180^c^ (140)	Shieh et al,^12^ (2020)	4658 (7622)	Latinx
WHI-AA	75 (67)	Allman et al,^13^ (2015)	7539 (416 cases)	African
WHI-LA	71 (66)		3363 (147 cases)	Latinx
ROOT	34 (31)	Wang et al,^14^ (2018)	3686 (1657)	African

Abbreviations: BCAC-L, Breast Cancer Association Consortium with large variant total; BCAC-S, Breast Cancer Association Consortium with small variant total; LATINAS, model with multiple cohorts of US Latina and Latin American women; ROOT, African Diaspora study; UKBB, UKBiobank; WHI-AA, Women’s Health Initiative for women with African ancestry; WHI-LA, Women’s Health Initiative for women with Latinx ancestry.

^a^ Variants overlap with the genotype data set in eMERGE.

^b^ All studies used independent GWAS data sets to develop PRS models.

^c^ While the original publication states there are 180 variants in the PRS, 1 variant was removed because of low imputation quality, which left 179 variants.

### Genotyping

Details of the eMERGE genotyping, imputation, and quality control procedures have been previously described.^8^ For this study, variants that match the following 3 criteria were retained for PRS calculation: (1) a mean *R^2^* imputation quality greater than 0.3 across genotype array-batches; (2) *P* value greater than 1 × 10^−6^ in ancestry-specific Hardy Weinberg Equilibrium tests; and (3) minor allele frequency (MAF) greater than 0.005. Principal component analysis was performed in both the combined data set and ancestry-specific data set after the MAF filtering and linkage disequilibrium (LD) pruning.

### Phenotyping

We used EMR data to phenotype each participant, including breast cancer case-control status, demographic information, ER status, family history, and age. We classified women as cases or controls using a validated phenotyping algorithm (above 95% positive predictive value for cases and negative predictive value for controls) that incorporated information from *International Classification of Diseases, Ninth Revision* (*ICD-9*) and *ICD-10* diagnostic codes (eTables 1 and2 in the Supplement), breast pathology reports, and medications (eTable 3 in the Supplement). The phenotyping workflow is shown in eFigure 1 in the Supplement, and more details can be found in the eMethods in the Supplement.

### Statistical Analysis

To evaluate the performance of each model, we standardized the PRSs to have a risk score unit expressed as an SD of the control distribution. The association of the standardized PRSs and breast cancer risk was evaluated by logistic regression adjusted for the first 3 ancestry-specific principal components,^8^ age, breast cancer family history, and study site. We defined age as the time period between the year the phenotyping algorithm was executed and the year of birth. In addition, we examined the association of breast cancer by percentiles of PRS, compared with the middle quantile (40% to 60%) or with the remainder of the population.

To examine the discrimination of each PRS, we estimated the area under the receiver operator characteristic curves (AUC), with only the PRS used as a predictor. To estimate the percentage of the total variance in breast cancer risk explained by PRS, we used Nagelkerke’s pseudo *R^2^* calculated for the full model inclusive of the PRS plus the covariates minus *R^2^* for the covariates alone. We also chose the PRS showing the largest effect size within each ancestry to estimate the cumulative risk of breast cancer for high PRS risk (top tertile), moderate PRS risk (middle tertile), and low risk (bottom tertile) individuals in each ancestry using iCARE^17^ (See eMethods in the Supplement).

We also assessed statistical power for testing associations of PRSs with breast cancer given sample size for each ancestry. Based on ancestry-specific empirical effect sizes of the PRS obtained from the literature, we assumed odds ratios (ORs) of 1.61,^2^ 1.23,^13^ and 1.58^12^ for women with European, African, and Latinx ancestry, respectively. Our power analysis shows we have 100%, 58%, and 99% power to detect an association with the above assumed ORs for women with European, African, and Latinx ancestries, respectively. When we assumed a moderate PRS effect size (OR, 1.39) for women with Latinx ancestry as reported in Allman et al,^13^ we observed 79% power to detect an association in Latinx women. However, if we assumed the same OR estimated for women with European ancestry in non-European women (ie, OR, 1.61), we should have 100% and 99% power to detect an association for breast cancer in women with African and Latinx ancestry, respectively. All analyses were carried out in R version 3.0.2 (R Project for Statistical Computing). All statistical tests were 2-sided, and *P* values < .05 were considered significant.

## Results

Our study included 39 591 women, including 33 594 women with European ancestry (mean [SD] age, 66.1 [17.7] years), 3801 with African ancestry (mean [SD] age, 59.6 [16.5] years), and 2196 with Latinx ancestry (mean [SD] age, 59.9 [19.4] years) (Table 2). The total number of variants included in the PRS calculation for each model is presented in Table 1.

**Table 2.** Participant Characteristics

Characteristic	Participants, No (%)		
	European ancestry, (n = 33 594)	African ancestry, (n = 3801)	Latinx ancestry, (n = 2196)
Age, mean (SD), y^a^	66.1 (17.7)	59.6 (16.5)	59.9 (19.4)
Breast cancer diagnosis	3960 (11.8)	274 (7.2)	147 (6.7)
Age at breast cancer diagnosis, mean (SD), y^b^	60.7 (13.0)	58.8 (12.5)	60.1 (13.0)
Estrogen receptor status, No. (% of cases)			
Positive	1052 (26.6)	20 (7.3)	22 (15.0)
Negative	241 (6.1)	15 (5.5)	4 (2.7)
Missing	2667 (67.3)	239 (87.2)	121 (82.3)
eMERGE network site			
Columbia University Medical Center	202 (0.6)	73 (1.9)	158 (7.2)
Geisinger	1330 (4)	4 (0.1)	8 (0.4)
Partners Healthcare	13 392 (39.9)	927 (24.4)	1093 (49.8)
Kaiser Permanente Washington Health Research Institute/University of Washington	1646 (4.9)	65 (1.7)	55 (2.5)
Mayo Clinic	3547 (10.6)	10 (0.3)	22 (1)
Marshfield Clinic Research Foundation	2815 (8.4)		8 (0.4)
Mount Sinai^c^	220 (0.7)	2515 (66.2)	742 (33.8)
Northwestern University	1878 (5.6)	207 (5.4)	23 (1)
Vanderbilt University	8564 (25.5)	0	87 (4)

Abbreviation: eMERGE, Electronic Medical Records and Genomics.

^a^ Age was calculated at the time of electronic phenotyping algorithm deployment.

^b^ Age at breast cancer diagnosis was defined as the age at the first breast cancer *International Classification of Diseases*–related code.

^c^ Mount Sinai only executed the phenotype algorithm for case-control definition; no estrogen receptor status data were extracted for this site.

### Association of PRS With Breast Cancer Risk in Women of European Ancestry

**Figure 1.**
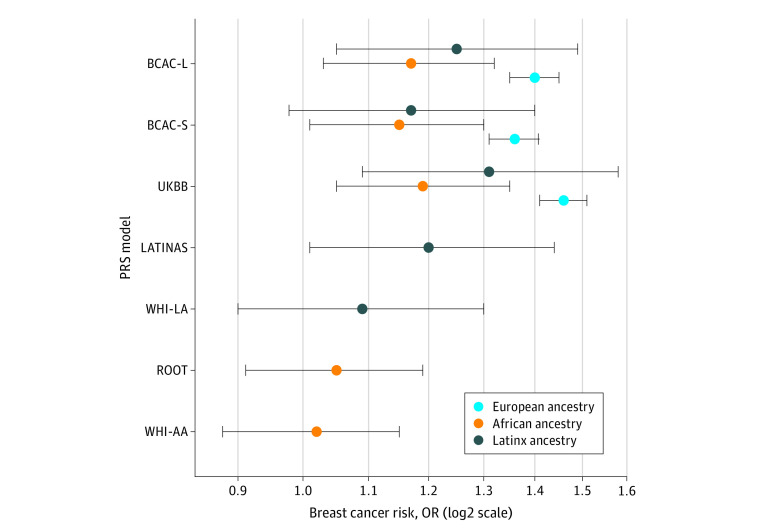
Association of Polygenic Risk Scores (PRSs) With Breast Cancer Risk in Women With European, African, and Latinx Ancestry in the eMERGE Cohorts Odds ratios (ORs) are adjusted for the first 3 ancestry-specific principal components, age, family history, and study site. Breast Cancer Association Consortium with small variant total (BCAC-S) includes 313 variants in the original PRS, BCAC with large variant total (BCAC-L) includes 3820 variants in the original PRS, Women’s Health Initiative for women with Latinx ancestry (WHI-LA) includes 71 variants in the original PRS and was optimized for women with Latinx ancestry, WHI for women with African ancestry (WHI-AA) includes 75 variants in the original PRS and was optimized for women with African ancestry, UKBiobank (UKBB) includes 5218 variants in the original PRS, African Diaspora study (ROOT) includes 34 variants in the original PRS and was optimized to women with African ancestry, and the LATINAS model includes 179 variants from multiple cohorts in the original PRS and was optimized for women with Latinx ancestry.

Our primary analysis examined the association of BCAC-S, BCAC-L, and UKBB in 3960 breast cancer cases and 29 634 control women with European ancestry and is shown in Figure 1. We found statistically significant associations with overall breast cancer risk for all 3 PRSs examined; with mean ORs per SD of the PRS ranging from 1.36 to 1.46, adjusted for the first 3 ancestry-specific principal components, age, family history, and study site (BCAC-L: OR, 1.40; 95% CI, 1.35-1.45; BCAC-S: OR, 1.36; 95% CI, 1.31-1.41; UKBB: OR, 1.46; 95% CI, 1.41-1.51).

**Figure 2.**
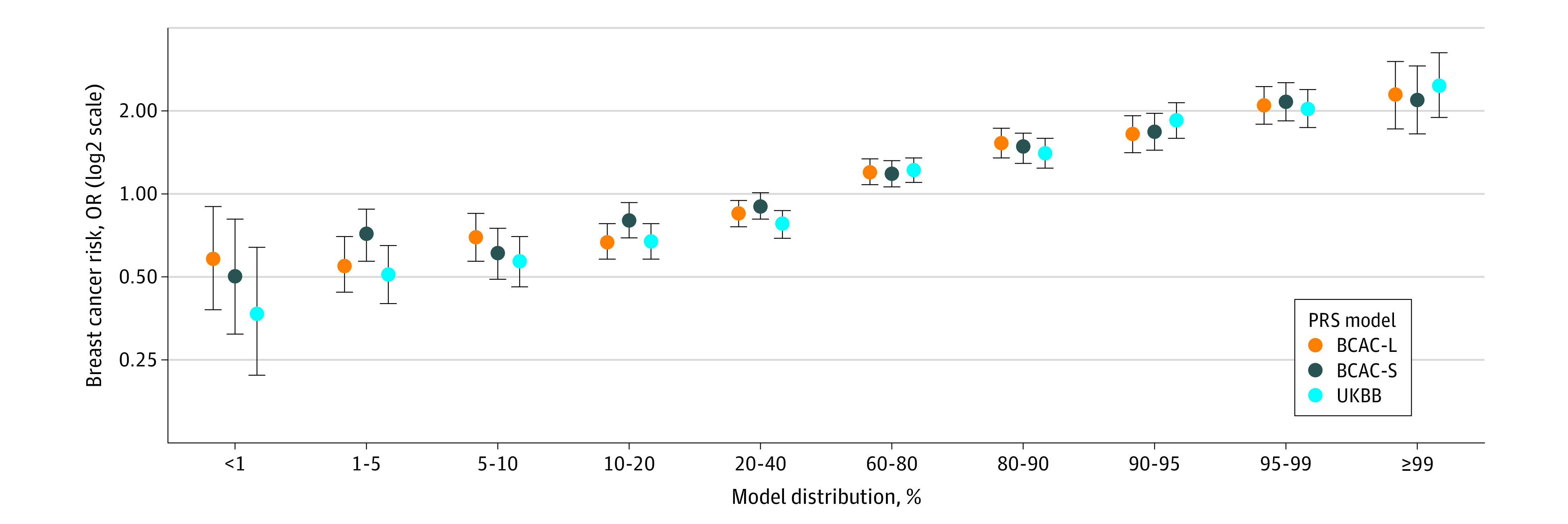
The Association of Polygenic Risk Scores (PRSs) With Overall Breast Cancer Risk in Women With European Ancestry Relative to the Middle Quantile Odds ratios (ORs) are adjusted for the first 3 ancestry-specific principal components, age, family history, and study site. BCAC-L indicates Breast Cancer Association Consortium with large variant total; BCAC-S, Breast Cancer Association Consortium with small variant total; UKBB, UKBiobank.

As illustrated in Figure 2, this association with breast cancer risk was largest in the extremes of the PRS distribution, with ORs ranging from 2.19 (95% CI, 1.84-2.53) to 2.48 (95% CI, 1.89-3.25) for the 3 different PRSs examined for those in the highest 1% of the PRS compared with those in the middle quantile. For example, for the UKBB PRS, we observed an approximate 2.5-fold increase in risk for those in the top 1% (OR, 2.48; 95% CI, 1.89-3.25) compared to those in the middle quantile (40%-60%) (Figure 2). Our findings were similar when we compared the extreme ends of the PRS distribution with those in the remainder of the PRS distribution (eFigure 2 in the Supplement). The AUCs were similar for all 3 PRSs (BCAC-L: AUC, 0.60; 95% CI, 0.59-0.61; BCAC-S: AUC, 0.59; 95% CI, 0.58-0.60; UKBB: AUC, 0.61; 95% CI, 0.60-0.62). The proportion of variance explained solely by PRS ranged from 1.7% to 2.5%, which is similar to what was reported originally (eg, 2.8% in the UKBB study^11^) (eTable 4 in the Supplement).

When we examined the association by ER status, we found significant associations for both ER-positive and ER-negative breast cancers, although the observed effect size was larger for ER-positive compared with ER-negative breast cancer (eFigure 3 in the Supplement). The findings were nearly identical for both overall PRSs and PRSs optimized for each breast cancer subtype (eFigure 3 in the Supplement).

### Association of PRS With Breast Cancer Risk in Women of African Ancestry

We examined the association of 5 previously developed PRSs: 3 based on women with European ancestry (BCAC-S, BCAC-L, and UKBB) and 2 developed in women of African ancestry (ROOT and WHI-AA) in 3801 women with African ancestry (including 274 cases). We found statistically significant associations for the 3 PRS models based on women with European ancestry and breast cancer risk with average ORs per SD of the PRS ranging from 1.15 (95% CI, 1.03-1.30) to 1.19 (95% CI, 1.04-1.35), but not for PRSs based on women with African ancestry (Figure 1). Compared with women with European ancestry, we observed lower AUCs in women with African ancestry (BCAC- L: AUC, 0.55; 95% CI, 0.51-0.58; BCAC-S: AUC, 0.53; 95% CI, 0.50-0.57; UKBB: AUC, 0.55; 95% CI, 0.52-0.59) (eTable 4 in the Supplement). The AUCs for PRSs developed in women with African ancestry were 0.52 (95% CI, 0.48-0.55) for ROOT and 0.50 (95% CI, 0.47-0.54) for WHI-AA.

### Association of PRS With Breast Cancer Risk in Latinx Women

We examined the association of 5 PRSs (BCAC-S, BCAC-L, UKBB, WHI-LA, and LATINAS), 2 of which were developed in or adapted to women with Latinx ancestry (WHI-LA and LATINAS) in 2196 Latinx women (including 147 cases). For Latinx women, we observed a statistically significant association for overall breast cancer risk for 3 of the PRSs examined (BCAC-L, UKBB, LATINAS), with ORs per SD ranging from 1.20 (95% CI, 1.01-1.42) to 1.31 (95% CI, 1.09-1.58) (Figure 1). Compared with women with European ancestry, we found lower AUCs in women with Latinx ancestry for BCAC-L, BCAC-S, and UKBB (with AUCs ranging from 0.53 to 0.56) (eTable 4 in the Supplement). The AUCs for PRSs developed in women with Latinx ancestry were 0.54 (95% CI, 0.47-0.62) for LATINAS and 0.48 (95% CI, 0.43-0.53) for WHI-LA.

### Estimation of Absolute Risk of Breast Cancer

**Figure 3.**
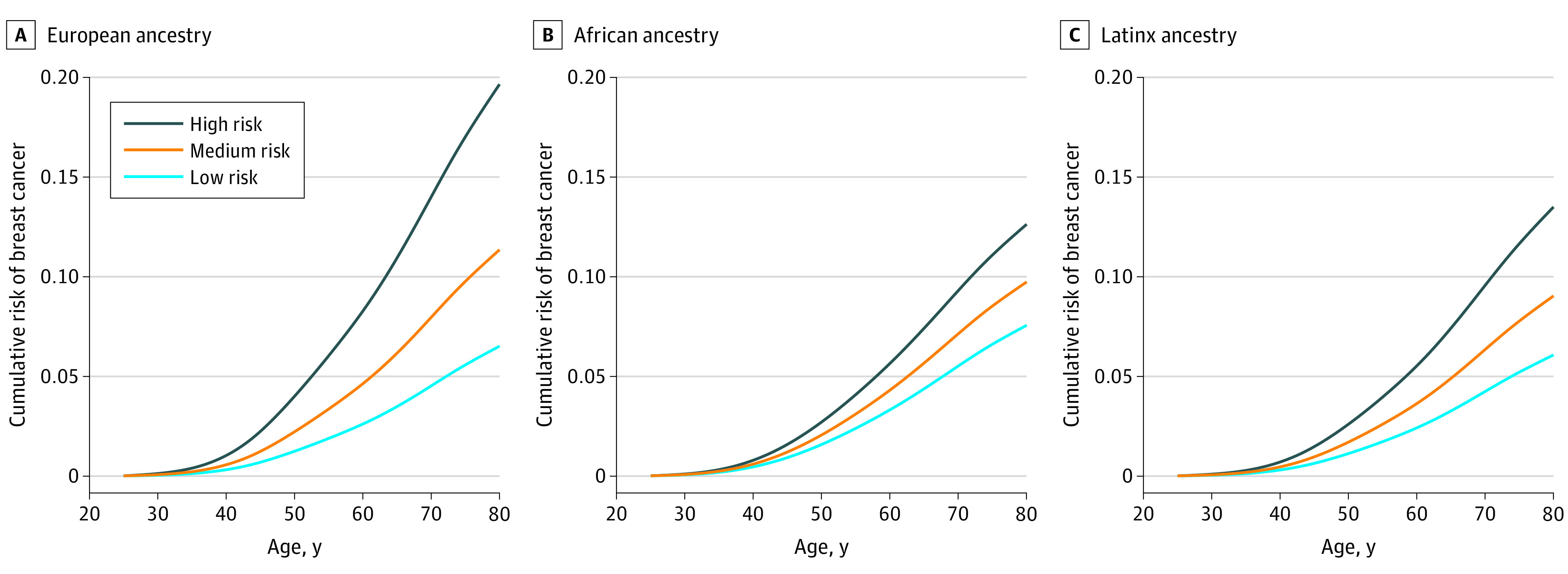
Cumulative Risk of Breast Cancer From Birth Estimated Using UKBB Polygenic Risk Score Model in Women With European, African, and Latinx Ancestry

As shown in Figure 3, there were differences in cumulative absolute breast cancer risk by risk categories of PRS for women with European, African, and Latinx ancestries when individuals were grouped into tertiles of the PRS distribution. When we compared those in the high PRS risk category with those at the low risk, women with European ancestry had larger risk gradients than women with African and Latinx ancestries. For example, women with European, African, and Latinx ancestries in the low PRS risk category had a cumulative breast cancer risk of 6.5%, 7.6%, and 6.1%, respectively, by age 80 years, whereas women in the high PRS risk category had 19.6%, 12.6% and 13.5% cumulative risk, respectively (Figure 3).

## Discussion

For PRSs developed in cohorts of women with European ancestry (UKBB, BCAC-L, BCAC-S), we replicated associations for increased breast cancer risk in women with European ancestry, although the ORs we observed in our study were smaller in magnitude than the original studies (eTable 5 in the Supplement). For example, BCAC-L had an OR of 1.40 in women with European ancestry compared with an OR of 1.66 (95% CI, 1.61-1.70) reported in the original study.^2^ This smaller magnitude might be explained by the reduced variant set, caused by the genotype platform discrepancy between the eMERGE network and published studies.

It is important to note that our study had limited sample size of women with non-European ancestries, despite using a large resource like eMERGE. Similar to other studies investigating the generalizability of PRSs in cohorts of women with European and non-European ancestry, we found that European ancestry–based PRS models generalized well in women with Latinx and African ancestry, but with attenuated associations observed in women with African ancestry as reported in a recent study.^18^ This is likely due to Latinx individuals in the US having a greater proportion of European ancestry than individuals with African ancestry.^19^ Previous work showed Latinx individuals in the eMERGE cohort have a complex genetic admixture with its principle component-based substructure centered mainly on the European samples with arms extending into the African and Asian groups.^8^ As such, the association detected for Latinx women in our cohort is likely driven by the proportion of underlying European ancestry. Future studies are needed to examine this association in different Latinx groups with greater African ancestry (eg, Caribbean groups) and Native American ancestry (eg, Central American groups).

Given the Eurocentricity of genomic studies, the smaller effect sizes for European ancestry–based PRSs with breast cancer risk in women with African ancestry in our study is not surprising and is consistent with PRS performance in non-European cohorts for other diseases^20,21,22,23^ and a large study examining European ancestry–based PRS in over 19 000 women with African ancestry, including over 9000 cases of breast cancer.^18^ While our power analysis suggests we have limited power (58%) to recover the signal detected by the original African ancestry–based PRS (OR, 1.23), we did have 100% power to detect an association in women with African ancestry if the European ancestry–based PRS can generalize as well in women with African ancestry (ie, if OR equaled or exceeded 1.61). The flip-flop phenomenon, in which a variant is a risk factor in 1 population but protective in another, has been observed among approximately 30% to 40% of variants across studies.^14^ Although the ROOT model used in our study only consisted of variants with the effect size in the same direction among women with European and African ancestries, it did not generalize well in the women with African ancestry in the eMERGE network. The poor generalizability may also be partly explained by differences in risk allele frequencies and LD patterns among diverse ancestries.^23^

Among the European ancestry–based PRSs (BCAC-S, BCAC-L, and UKBB), UKBB achieved the largest effect size in the eMERGE cohort among women with European ancestry. Although UKBB used the same genome-wide association study (GWAS) summary statistics provided in the BCAC study, it developed and validated the PRS based on an independent larger sample size collected through UK Biobank, which can contribute to its stronger generalizability. Another possible explanation is that UKBB’s similar phenotype definition and data was collected in the clinical setting utilizing EMRs. However, our phenotype algorithm included women with ductal carcinoma in situ (DCIS) who have stage 0 or noninvasive breast cancer. Our sensitivity analysis suggested that defining cases excluding DCIS achieved a slightly higher OR (eTable 6 in the Supplement). Because DCIS cases often requires definitive treatment with complete surgical resection, radiation therapy, and adjuvant hormonal therapy, we believe a validated PRS should also be able to make prediction for DCIS cases. Of note, some breast cancer risk prediction tools such as the Tyrer-Cuzick model^24^ account for both invasive and noninvasive breast cancer. Future PRS development work may consider including DCIS in the training sample.

For PRSs developed in non-European ancestry study populations (WHI-AA, WHI-LA, and ROOT) or adapted to non-European ancestry populations (LATINAS), we did not replicate the previously reported associations in the eMERGE cohort for women with Latinx or African ancestry, except for the LATINAS in Latinx women. LATINAS is a multiethnic PRS that utilized effect sizes obtained from populations with European ancestry and further developed the PRS in a cohort of Latinx women, suggesting that combining training data from samples from individuals with European ancestry could improve the observed associations in non-European ancestry populations.^25,26^ We found that while 61 of 179 variants (34.1%) included in LATINAS were also included in UKBB model, only 12 of 71 (16.9%) included in WHI-LA were included in the UKBB model. Because the PRSs developed in studies using populations of non-European ancestry are often based on much smaller GWAS cohorts, the uncertainty of the effect sizes used in those PRSs is larger, making their predictive power lower for populations with non-European ancestry.^12,14^ In addition, the PRSs based on individuals with non-European ancestry included fewer variants passing the statistical threshold because of the smaller sample size in the discovery GWAS cohort, which would possibly contribute further to their weaker generalizability. Of note is the limited sample size for women with Latinx or African ancestry in our study, so future studies with adequate power are warranted to evaluate PRS performance for these groups. Furthermore, even with an adequate sample size for populations with non-European ancestry, limitations inherent to the genotyping platforms used in GWAS^27^ can make this subpopulation optimization theoretically insufficient to reduce the bias if the subpopulation risk allele is not captured by the genotype platform, which is possible because many array designs are based on samples of populations with European ancestry. Moreover, previous findings that women with African ancestry have a 40% higher mortality rate,^28^ which is often attributed to later stage of diagnosis and related preventative health care barriers, underscores the urgent need to increase diversity in genomic studies so that future clinical applications of the PRS do not exacerbate existing health disparities.

The eMERGE^29^ and the All of Us Research Program^30^ are 2 programs actively involved in increasing recruitment of diverse patients to help address the gap. These EMR-derived cohorts provide a scalable approach to independently validate previously developed PRSs for different phenotypes across multiple clinical operation sites.^31,32,33^ We found similar magnitudes of PRS association in women with European ancestry across all study sites, except for Vanderbilt University (eFigure 4 in the Supplement). This difference might be related to the heterogeneity in the genotyping platforms and/or EMR systems.^34,35^ Breast cancer PRS models based on populations with non-European ancestry are still in development via large consortia studies, such as the Confluence Project,^36^ which aims to develop a large research resource including at least 300 000 breast cancer cases and 300 000 controls of different races/ethnicities by the confluence of existing GWAS and new genome-wide genotyping data

### Limitations

This study had several limitations. The small sample size of women with Latinx or African ancestry in our study is a limitation, particularly in being able to examine associations for women at the extreme ends of the PRS and by BC subtype. Missing marker information was much more common in women in these groups than for women with European ancestry and imputation is generally poorer in populations with non-European ancestry, potentially leading to important and unmeasurable biases. Also, while the eMERGE network is a rich and unique resource for this study, it is primarily focused on academic centers, and may not be generalizable to patients in community practices. Additionally, our validation is based on PRSs constructed using a reduced variant set because of the genotype platform discrepancy between the eMERGE network and published studies. A variant in the original model can be excluded for multiple reasons such as ambiguity (ie, those with complementary alleles, either C/G or A/T), low imputation quality, or allele mismatch. Theoretically, expected PRSs can be calculated for the full variant set in the original published PRS by taking the imputed probabilities for mismatched genotypes into consideration. However, given the low imputation quality for those mismatched genotypes we excluded in this study, the expected PRS for the full variant set could have a large variance, and as such, we did not conduct the calculation in our study. Our sensitivity analysis found that while using a more conservative imputation quality threshold (ie, imputation *R^2^* > 0.8) significantly reduced the number of variants in the genotype data set, our results were largely unchanged (eFigure 5 and eTable 7 in the Supplement).

## Conclusions

In summary, we found PRS models based on populations with European ancestry were significantly associated with breast cancer risk in women with European ancestry in the eMERGE network. We also found that these PRSs generalized well to women with European and Latinx ancestry, and to a lesser degree to women with African ancestry, although further studies with larger sample size of women with African ancestry are needed. Additionally, we found that PRS developed in small GWAS studies of populations with non-European ancestry did not generalize well in the respective ancestry group. Our results highlight the need to increase the inclusion of racially and ethnically diverse individuals, particularly individuals with African ancestry, in large-scale genomic studies. Until well-developed and validated PRSs for women with non-European ancestry become available, the current PRSs developed based on cohorts with European ancestry could be adapted for Latinx women, but not women with African ancestry, in clinical settings until additional data sets become available in this important and high-risk group.

## Notes

Supplement. **eTable 1.** Female Breast Cancer Diagnostic Codes **eTable 2.** Breast Cancer History Codes **eTable 3.** Hormone Therapy Drugs for Breast Cancer **eTable 4.** The AUC (95% CI) for Breast Cancer Prediction Using PRS as a Single Predictor in Women of Different Ancestries **eTable 5.** Associations of PRS With Breast Cancer in the Original Studies for EA Women **eTable 6.** The OR (95% CI) of Breast Cancer per Standard PRS Unit Increase for Women of Different Ancestries in the eMERGE Cohorts **eTable 7.** The Different Factors Can Affect the Reducing of the Number of SNPs **eFigure 1.** Breast Cancer Phenotyping Workflow **eFigure 2.** The Association of Overall Breast Cancer (BC) for European Ancestry Women in the Highest 20%, 10%, 5%, and 1% of PRS Risk, Compared to Women With Lower PRS Risk (PRS Percentiles <80, 90, 95, and 99, Respectively) **eFigure 3.** The Association Between Different PRSs and Estrogen Receptor (ER)-positive and ER-negative Breast Cancer for Women of European Ancestry **eFigure 4.** The Association of the Different PRSs and Breast Cancer (BC) Risk in Women of European, African, and Latinx Ancestries, Stratified by eMERGE Study Sites **eFigure 5.** The Association Between Different PRSs Generated by Various Filter Stacks and Overall Breast Cancer for Women eMethods. eReferences.
