# Supplementary material for: Generalizability of Polygenic Risk Scores for Breast Cancer Among Women With European, African, and Latinx Ancestry
Source: JAMA Netw Open. 2021 Aug 4;4(8):e2119084. doi: 10.1001/jamanetworkopen.2021.19084 (PMC8339934; doi:10.1001/jamanetworkopen.2021.19084)
Supplement: Supplement. — eTable 1. Female Breast Cancer Diagnostic Codes eTable 2. Breast Cancer History Codes eTable 3. Hormone Therapy Drugs for Breast Cancer eTable 4. The AUC (95% CI) for Breast Cancer Prediction Using PRS as a Single Predictor in Women of Different Ancestries eTable 5. Associations of PRS With Breast Cancer in the Original Studies for EA Women eTable 6. The OR (95% CI) of Breast Cancer per Standard PRS Unit Increase for Women of Different Ancestries in the eMERGE Cohorts eTable 7. The Different Factors Can Affect the Reducing of the Number of SNPs eFigure 1. Breast Cancer Phenotyping Workflow eFigure 2. The Association of Overall Breast Cancer (BC) for European Ancestry Women in the Highest 20%, 10%, 5%, and 1% of PRS Risk, Compared to Women With Lower PRS Risk (PRS Percentiles <80, 90, 95, and 99, Respectively) eFigure 3. The Association Between Different PRSs and Estrogen Receptor (ER)-positive and ER-negative Breast Cancer for Women of European Ancestry eFigure 4. The Association of the Different PRSs and Breast Cancer (BC) Risk in Women of European, African, and Latinx Ancestries, Stratified by eMERGE Study Sites eFigure 5. The Association Between Different PRSs Generated by Various Filter Stacks and Overall Breast Cancer for Women eMethods. eReferences. [file jamanetwopen-e2119084-s001.pdf]

Supplemental Online Content

Liu C, Zeinomar N, Chung WK, et al. Generalizability of polygenic risk scores for breast cancer among women with European, African, and Latinx ancestry. *JAMA Netw Open.* 2021;4(8):e2119084.
doi:10.1001/jamanetworkopen.2021.19084

eTable 1. Female Breast Cancer Diagnostic Codes

eTable 2. Breast Cancer History Codes

eTable 3. Hormone Therapy Drugs for Breast Cancer

eTable 4. The AUC (95% CI) for Breast Cancer Prediction Using PRS as a Single Predictor in Women of Different Ancestries

eTable 5. Associations of PRS With Breast Cancer in the Original Studies for EA Women

eTable 6. The OR (95% CI) of Breast Cancer per Standard PRS Unit Increase for Women of Different Ancestries in the eMERGE Cohorts

eTable 7. The Different Factors Can Affect the Reducing of the Number of SNPs

eFigure 1. Breast Cancer Phenotyping Workflow

eFigure 2. The Association of Overall Breast Cancer (BC) for European Ancestry Women in the Highest 20%, 10%, 5%, and 1% of PRS Risk, Compared to Women With Lower PRS Risk (PRS Percentiles <80, 90, 95, and 99, Respectively)

eFigure 3. The Association Between Different PRSs and Estrogen Receptor (ER)-positive and ER-negative Breast Cancer for Women of European Ancestry

© 2021 Liu C et al. *JAMA Network Open.*

eFigure 4. The Association of the Different PRSs and Breast Cancer (BC) Risk in Women of European, African, and Latinx Ancestries, Stratified by eMERGE Study Sites

eFigure 5. The Association Between Different PRSs Generated by Various Filter Stacks and Overall Breast Cancer for Women

eMethods.

eReferences.

This supplemental material has been provided by the authors to give readers additional information about their work.

© 2021 Liu C et al. *JAMA Network Open.*

eTable 1. Female breast cancer diagnostic codes.

OMOP Domain[a]	Vocabulary	ICD code	ICD name	Standard OMOP ID	Standard OMOP name
Condition	ICD10CM	C50.01	Malignant neoplasm of nipple and areola, female	4091464	Malignant neoplasm of nipple and areola of female breast
Condition	ICD10CM	C50.011	Malignant neoplasm of nipple and areola, right female breast	4091464	Malignant neoplasm of nipple and areola of female breast
Condition	ICD10CM	C50.012	Malignant neoplasm of nipple and areola, left female breast	4091464	Malignant neoplasm of nipple and areola of female breast
Condition	ICD10CM	C50.019	Malignant neoplasm of nipple and areola, unspecified female breast	4091464	Malignant neoplasm of nipple and areola of female breast
Condition	ICD10CM	C50.1	Malignant neoplasm of central portion of breast	4092511	Malignant neoplasm of central part of female breast
Condition	ICD10CM	C50.11	Malignant neoplasm of central portion of breast, female	4092511	Malignant neoplasm of central part of female breast
Condition	ICD10CM	C50.111	Malignant neoplasm of central portion of right female breast	4092511	Malignant neoplasm of central part of female breast

© 2021 Liu C et al. *JAMA Network Open.*

OMOP Domain[a]	Vocabulary	ICD code	ICD name	Standard OMOP ID	Standard OMOP name
Condition	ICD10CM	C50.112	Malignant neoplasm of central portion of left female breast	4092511	Malignant neoplasm of central part of female breast
Condition	ICD10CM	C50.119	Malignant neoplasm of central portion of unspecified female breast	4092511	Malignant neoplasm of central part of female breast
Condition	ICD10CM	C50.2	Malignant neoplasm of upper-inner quadrant of breast	4187849	Malignant neoplasm of breast upper inner quadrant
Condition	ICD10CM	C50.21	Malignant neoplasm of upper-inner quadrant of breast, female	4092512	Malignant neoplasm of upper-inner quadrant of female breast
Condition	ICD10CM	C50.211	Malignant neoplasm of upper-inner quadrant of right female breast	4092512	Malignant neoplasm of upper-inner quadrant of female breast
Condition	ICD10CM	C50.212	Malignant neoplasm of upper-inner quadrant of left female breast	4092512	Malignant neoplasm of upper-inner quadrant of female breast
Condition	ICD10CM	C50.219	Malignant neoplasm of upper-inner quadrant of unspecified female breast	4092512	Malignant neoplasm of upper-inner quadrant of female breast
Condition	ICD10CM	C50.3	Malignant neoplasm of lower-inner quadrant of breast	4188544	Malignant neoplasm of breast lower inner quadrant
Condition	ICD10CM	C50.31	Malignant neoplasm of lower-inner quadrant of breast, female	4095740	Malignant neoplasm of lower-inner quadrant of female breast

© 2021 Liu C et al. *JAMA Network Open.*

OMOP Domain[a]	Vocabulary	ICD code	ICD name	Standard OMOP ID	Standard OMOP name
Condition	ICD10CM	C50.311	Malignant neoplasm of lower-inner quadrant of right female breast	4095740	Malignant neoplasm of lower-inner quadrant of female breast
Condition	ICD10CM	C50.312	Malignant neoplasm of lower-inner quadrant of left female breast	4095740	Malignant neoplasm of lower-inner quadrant of female breast
Condition	ICD10CM	C50.319	Malignant neoplasm of lower-inner quadrant of unspecified female breast	4095740	Malignant neoplasm of lower-inner quadrant of female breast
Condition	ICD10CM	C50.4	Malignant neoplasm of upper-outer quadrant of breast	4160780	Malignant neoplasm of breast upper outer quadrant
Condition	ICD10CM	C50.41	Malignant neoplasm of upper-outer quadrant of breast, female	4091465	Malignant neoplasm of upper-outer quadrant of female breast
Condition	ICD10CM	C50.411	Malignant neoplasm of upper-outer quadrant of right female breast	4091465	Malignant neoplasm of upper-outer quadrant of female breast
Condition	ICD10CM	C50.412	Malignant neoplasm of upper-outer quadrant of left female breast	4091465	Malignant neoplasm of upper-outer quadrant of female breast
Condition	ICD10CM	C50.419	Malignant neoplasm of upper-outer quadrant of unspecified female breast	4091465	Malignant neoplasm of upper-outer quadrant of female breast
Condition	ICD10CM	C50.5	Malignant neoplasm of lower-outer quadrant of breast	4187848	Malignant neoplasm of breast lower outer quadrant

© 2021 Liu C et al. *JAMA Network Open.*

OMOP Domain[a]	Vocabulary	ICD code	ICD name	Standard OMOP ID	Standard OMOP name
Condition	ICD10CM	C50.51	Malignant neoplasm of lower-outer quadrant of breast, female	4091466	Malignant neoplasm of lower-outer quadrant of female breast
Condition	ICD10CM	C50.511	Malignant neoplasm of lower-outer quadrant of right female breast	4091466	Malignant neoplasm of lower-outer quadrant of female breast
Condition	ICD10CM	C50.512	Malignant neoplasm of lower-outer quadrant of left female breast	4091466	Malignant neoplasm of lower-outer quadrant of female breast
Condition	ICD10CM	C50.519	Malignant neoplasm of lower-outer quadrant of unspecified female breast	4091466	Malignant neoplasm of lower-outer quadrant of female breast
Condition	ICD10CM	C50.6	Malignant neoplasm of axillary tail of breast	4155292	Malignant neoplasm of axillary tail of breast
Condition	ICD10CM	C50.61	Malignant neoplasm of axillary tail of breast, female	4091467	Malignant neoplasm of axillary tail of female breast
Condition	ICD10CM	C50.611	Malignant neoplasm of axillary tail of right female breast	4091467	Malignant neoplasm of axillary tail of female breast
Condition	ICD10CM	C50.612	Malignant neoplasm of axillary tail of left female breast	4091467	Malignant neoplasm of axillary tail of female breast
Condition	ICD10CM	C50.619	Malignant neoplasm of axillary tail of unspecified female breast	4091467	Malignant neoplasm of axillary tail of female breast

OMOP Domain[a]	Vocabulary	ICD code	ICD name	Standard OMOP ID	Standard OMOP name
Condition	ICD10CM	C50.81	Malignant neoplasm of overlapping sites of breast, female	133711	Overlapping malignant neoplasm of female breast
Condition	ICD10CM	C50.811	Malignant neoplasm of overlapping sites of right female breast	133711	Overlapping malignant neoplasm of female breast
Condition	ICD10CM	C50.812	Malignant neoplasm of overlapping sites of left female breast	133711	Overlapping malignant neoplasm of female breast
Condition	ICD10CM	C50.819	Malignant neoplasm of overlapping sites of unspecified female breast	133711	Overlapping malignant neoplasm of female breast
Condition	ICD10CM	C50.91	Malignant neoplasm of breast of unspecified site, female	4157332	Malignant neoplasm of female breast
Condition	ICD10CM	C50.911	Malignant neoplasm of unspecified site of right female breast	4157332	Malignant neoplasm of female breast
Condition	ICD10CM	C50.912	Malignant neoplasm of unspecified site of left female breast	4157332	Malignant neoplasm of female breast
Condition	ICD10CM	C50.919	Malignant neoplasm of unspecified site of unspecified female breast	4157332	Malignant neoplasm of female breast
Condition	ICD10CM	D05	Carcinoma in situ of breast	81250	Carcinoma in situ of breast
Condition	ICD10CM	D05.1	Intraductal carcinoma in situ of breast	4001670	Intraductal carcinoma in situ of breast

© 2021 Liu C et al. *JAMA Network Open.*

OMOP Domain[a]	Vocabulary	ICD code	ICD name	Standard OMOP ID	Standard OMOP name
Condition	ICD10CM	D05.10	Intraductal carcinoma in situ of unspecified breast	4001670	Intraductal carcinoma in situ of breast
Condition	ICD10CM	D05.11	Intraductal carcinoma in situ of right breast	4001670	Intraductal carcinoma in situ of breast
Condition	ICD10CM	D05.12	Intraductal carcinoma in situ of left breast	4001670	Intraductal carcinoma in situ of breast
Condition	ICD10CM	D05.8	Other specified type of carcinoma in situ of breast	81250	Carcinoma in situ of breast
Condition	ICD10CM	D05.80	Other specified type of carcinoma in situ of unspecified breast	81250	Carcinoma in situ of breast
Condition	ICD10CM	D05.81	Other specified type of carcinoma in situ of right breast	81250	Carcinoma in situ of breast
Condition	ICD10CM	D05.82	Other specified type of carcinoma in situ of left breast	81250	Carcinoma in situ of breast
Condition	ICD10CM	D05.9	Unspecified type of carcinoma in situ of breast	81250	Carcinoma in situ of breast
Condition	ICD10CM	D05.90	Unspecified type of carcinoma in situ of unspecified breast	81250	Carcinoma in situ of breast

© 2021 Liu C et al. *JAMA Network Open.*

OMOP Domain[a]	Vocabulary	ICD code	ICD name	Standard OMOP ID	Standard OMOP name
Condition	ICD10CM	D05.91	Unspecified type of carcinoma in situ of right breast	81250	Carcinoma in situ of breast
Condition	ICD10CM	D05.92	Unspecified type of carcinoma in situ of left breast	81250	Carcinoma in situ of breast
Condition	ICD9CM	174	Malignant neoplasm of female breast	4157332	Malignant neoplasm of female breast
Condition	ICD9CM	174.0	Malignant neoplasm of nipple and areola of female breast	4091464	Malignant neoplasm of nipple and areola of female breast
Condition	ICD9CM	174.1	Malignant neoplasm of central portion of female breast	432845	Primary malignant neoplasm of central portion of female breast
Condition	ICD9CM	174.2	Malignant neoplasm of upper-inner quadrant of female breast	4092512	Malignant neoplasm of upper-inner quadrant of female breast
Condition	ICD9CM	174.3	Malignant neoplasm of lower-inner quadrant of female breast	4095740	Malignant neoplasm of lower-inner quadrant of female breast
Condition	ICD9CM	174.4	Malignant neoplasm of upper-outer quadrant of female breast	4091465	Malignant neoplasm of upper-outer quadrant of female breast
Condition	ICD9CM	174.5	Malignant neoplasm of lower-outer quadrant of female breast	4091466	Malignant neoplasm of lower-outer quadrant of female breast
Condition	ICD9CM	174.6	Malignant neoplasm of axillary tail of female breast	4091467	Malignant neoplasm of axillary tail of female breast

© 2021 Liu C et al. *JAMA Network Open.*

OMOP Domain[a]	Vocabulary	ICD code	ICD name	Standard OMOP ID	Standard OMOP name
Condition	ICD9CM	174.8	Malignant neoplasm of other specified sites of female breast	133711	Overlapping malignant neoplasm of female breast
Condition	ICD9CM	174.9	Malignant neoplasm of breast (female), unspecified	137809	Primary malignant neoplasm of female breast
Condition	ICD9CM	233.0	Carcinoma in situ of breast	81250	Carcinoma in situ of breast
Observation	ICD10CM	Z85.3	Personal history of malignant neoplasm of breast	4214956	History of clinical finding in subject
Observation	ICD10CM	Z86.000	Personal history of in-situ neoplasm of breast	4214956	History of clinical finding in subject
Observation	ICD9CM	V10.3	Personal history of malignant neoplasm of breast	4214956	History of clinical finding in subject

[a] **OMOP**: The Observational Medical Outcomes Partnership (OMOP) common data model.

© 2021 Liu C et al. *JAMA Network Open.*

eTable 2. Breast cancer history codes

OMOP Domain[a]	Vocabulary	ICD Code	ICD Name	Standard OMOP ID	Standard OMOP Name
Observation	ICD10CM	Z85.3	Personal history of malignant neoplasm of breast	4214956	History of clinical finding in subject
Observation	ICD10CM	Z86.000	Personal history of in-situ neoplasm of breast	4214956	History of clinical finding in subject
Observation	ICD9CM	V10.3	Personal history of malignant neoplasm of breast	4214956	History of clinical finding in subject

[a] **OMOP**: The Observational Medical Outcomes Partnership (OMOP) common data model.

© 2021 Liu C et al. *JAMA Network Open.*

eTable 3. Hormone therapy drugs for breast cancer

Drug (Brand Name)	Drug Name	RxCUI	Standard OMOP ID
Tamoxifen (Nolvadex)	Tamoxifen	10324	1436678
	Nolvadex	281964	19004028
Anastrozole (Arimidex)	Anastrozole	84857	1348265
	Arimidex	262485	19100842
Letrozole (Femara)	Letrozole	72965	1315946
	Femara	203769	19048060
Exemestane (Aromasin)	Exemestane	258494	1398399
	Aromasin	262105	19100386
Goserelin (Zoladex)	Goserelin	50610	1366310
	Zoladex	58328	19079531
Leuprolide (Lupron)	Leuprolide	42375	1351541
	Lupron	203852	19048354
Fulvestrant (Faslodex)	Fulvestrant	282357	1304044
	Faslodex	203870	19048400
Toremifene (Fareston)	Toremifene	38409	1342346
	Fareston	155082	19024254

© 2021 Liu C et al. *JAMA Network Open.*

eTable 4. The AUC (95% CI) for breast cancer prediction using PRS as a single predictor in women of different ancestries

PRS	**EA** (3960 breast cancer cases/ 29634 controls)		**AA** (274 breast cancer cases/ 3527 controls)		**LA** (147 breast cancer cases/ 2049 controls)	
	AUC (95% CI)	R^2 (%)[a]	AUC (95% CI)	R^2 (%)	AUC (95% CI)	R^2 (%)
BCAC-L	0.60 (0.59-0.61)	2.5	0.55 (0.51-0.58)	0.3	0.55 (0.50-0.60)	0.8
BCAC-S	0.59 (0.58-0.60)	2.1	0.53 (0.50-0.57)	0.2	0.53 (0.48-0.58)	0.4
UKBB	0.61 (0.60-0.62)	1.7	0.55 (0.52-0.59)	0.4	0.56 (0.51-0.60)	1.0
LATINAS	-	-	-	-	0.54 (0.47-0.62)	0.5
ROOT	-	-	0.52 (0.48-0.55)	<0.1	-	-
WHI-AA	-	-	0.50 (0.47-0.54)	<0.1	-	-
WHI-LA	-	-	-	-	0.48 (0.43-0.53)	0.1

[a]Nagelkerke's pseudo R^2 calculated for the full model inclusive of the PRS plus the covariates minus R^2 for the covariates alone.

© 2021 Liu C et al. *JAMA Network Open.*

eTable 5. Associations of PRS with breast cancer in the original studies for EA women

PRS Model	Odds ratio (95% Confidence Interval)	
	eMERGE	Original study
**BCAC-L** [1]	1.4 (1.35-1.45)	1.66 (1.61-1.70)
**BCAC-S** [1]	1.36 (1.31-1.41)	1.61 (1.57-1.65)
**UKBB** [2]	1.46 (1.41-1.51)	N/A

© 2021 Liu C et al. *JAMA Network Open.*

eTable 6. The OR (95% CI) of breast cancer per standard PRS unit increase for women of different ancestries in the eMERGE cohorts

PRS Model	African ancestry (231/3527)	European ancestry (3345 / 29634)	LatinX ancestry (127 / 2049)
**BCAC-L**	1.26 (1.1,1.44)	1.42 (1.37,1.48)	1.3 (1.08,1.57)
**BCAC-S**	1.21 (1.06,1.39)	1.37 (1.32,1.42)	1.18 (0.97,1.42)
**LATINAS**	N/A	N/A	1.2 (1,1.46)
**ROOT**	1.08 (0.94,1.24)	N/A	N/A
**UKBB**	1.24 (1.08,1.43)	1.47 (1.42,1.53)	1.35 (1.11,1.64)
**WHI-AA**	1.05 (0.92,1.21)	N/A	N/A
**WHI-LA**	N/A	N/A	1.09 (0.9,1.32)

Breast cancer case and controls are defined according to ICD codes used in Khera et al. [2] (excluding DCIS).

© 2021 Liu C et al. *JAMA Network Open.*

eTable 7. The different factors can affect the reducing of the number of SNPs

PRS Models	BCAC-S	BCAC-L	WHI-LA	WHI-AA	UKBB	ROOT	LATINAS
# of SNPs in original model	313	3820	71	75	5218	34	180(179)
# of SNP left after apply a single filter							
no ambiguous	282	2535	66	68	4504	31	140
no allele mismatch	240	2535	66	68	4504	31	140
no 3+allele	312	2535	66	68	4504	31	140
Genotyped or imputed in eMERGE	241	2535	66	68	4504	31	140
imputated Rsq>0.3	241	2534	66	68	4447	31	140
imputated Rsq>0.8	225	2236	65	68	2805	31	132
HWE>1e-6 in ANY ancestry	241	2535	66	68	4504	31	140
HWE>1e-6 in ALL ancestry	238	2511	64	67	4449	30	135
MAF>0.005	240	2533	66	68	4209	31	140
MAF>0.01	238	2493	65	68	3782	31	139
# of SNPs in the final model							
# of SNPs in filter stack 1	210	2535	66	68	4504	31	140
# of SNPs in filter stack 2	209	2532	66	67	4192	31	140
# of SNPs in filter stack 3	193	2213	64	66	2768	30	129

Filter stack 1: no allele mismatch, no 3+allele, no 3+allele, and Genotyped or imputed in eMERGE; Filter stack 2: no allele mismatch, no 3+allele, no 3+allele, Genotyped or imputed in eMERGE, imputated Rsq>0.3, HWE>1e-6 in ANY ancestry, and MAF>0.005; Filter stack 3: no allele mismatch, no 3+allele, no 3+allele, Genotyped or imputed in eMERGE, imputated Rsq>0.8, HWE>1e-6 in ALL ancestry, and MAF>0.01;

© 2021 Liu C et al. *JAMA Network Open.*

eFigure1. Breast cancer phenotyping workflow. In the breast cancer (BC) phenotyping algorithm the hormone receptor status were extracted for estrogen receptor (ER) and progesterone receptor (PR) BC. ER determined subtypes (i.e. ER-positive or ER-negative) were used for analysis. Mount Sinai only provided the case control definition without further subtype information extracted.

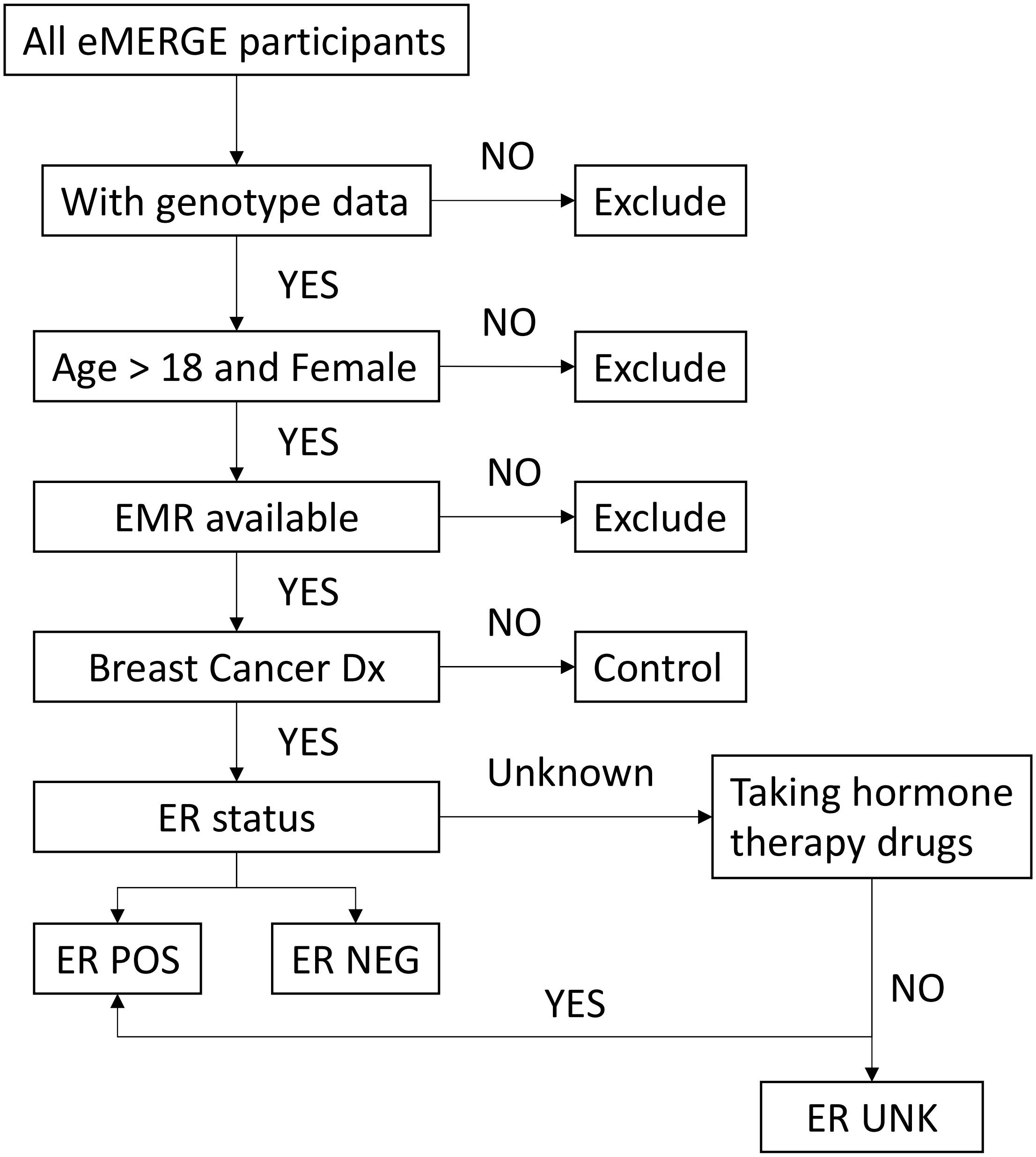

© 2021 Liu C et al. *JAMA Network Open.*

eFigure 2. The association of overall breast cancer (BC) for European ancestry women in the highest 20%, 10%, 5%, and 1% of PRS risk, compared to women with lower PRS risk (PRS percentiles <80, 90, 95, and 99, respectively)

Odds ratios and 95% confidence intervals, adjusted for the first 3 ancestry-specific principal components, age, family history, and study site are shown.

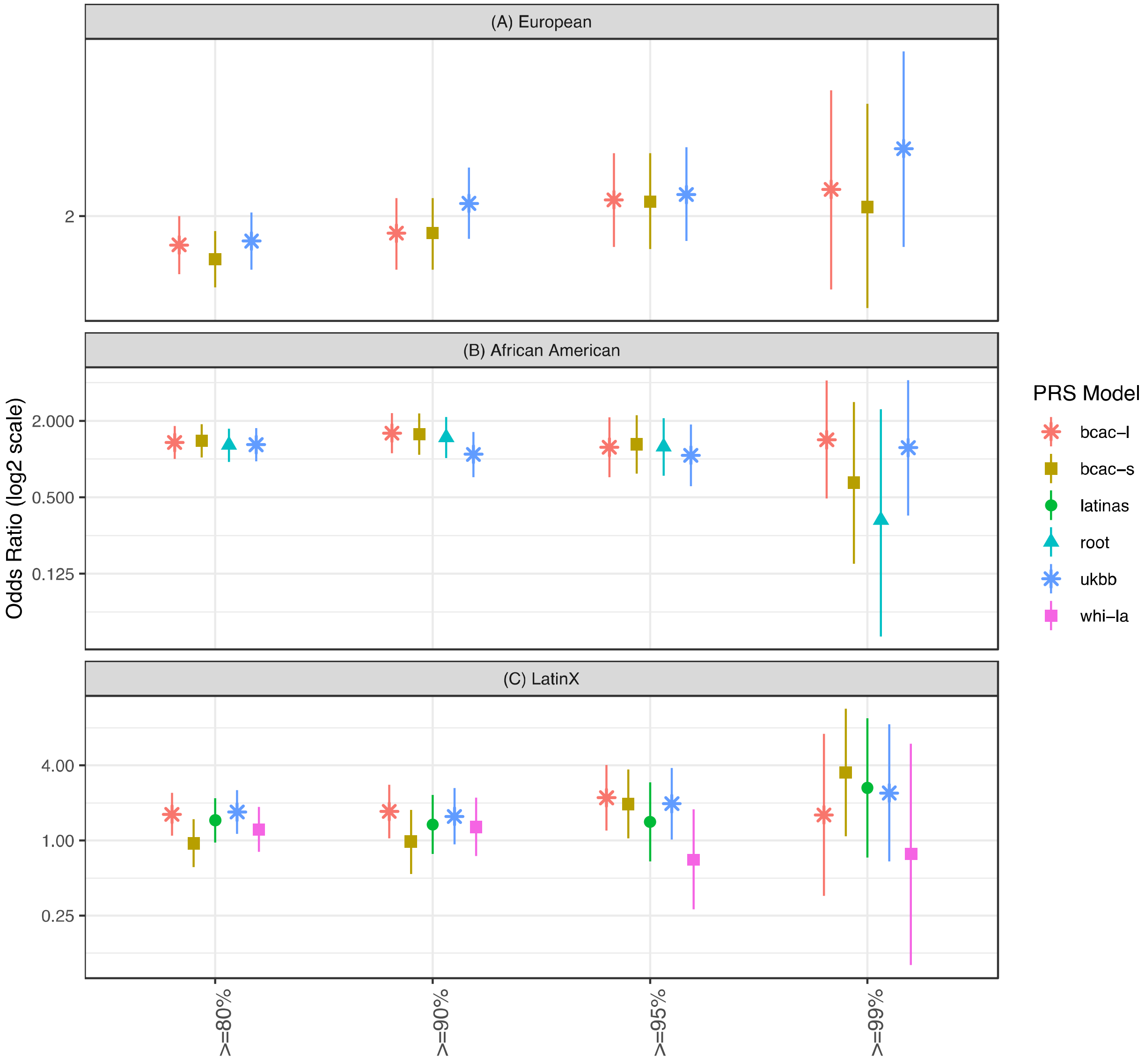

© 2021 Liu C et al. *JAMA Network Open.*

eFigure 3. The association between different PRSs and estrogen receptor (ER)-positive and ER-negative breast cancer for women of European ancestry

Odds ratios and 95% confidence intervals are presented for each of the of breast cancer subtypes (A: ER-positive and B: ER-negative) per standard PRS unit increase.

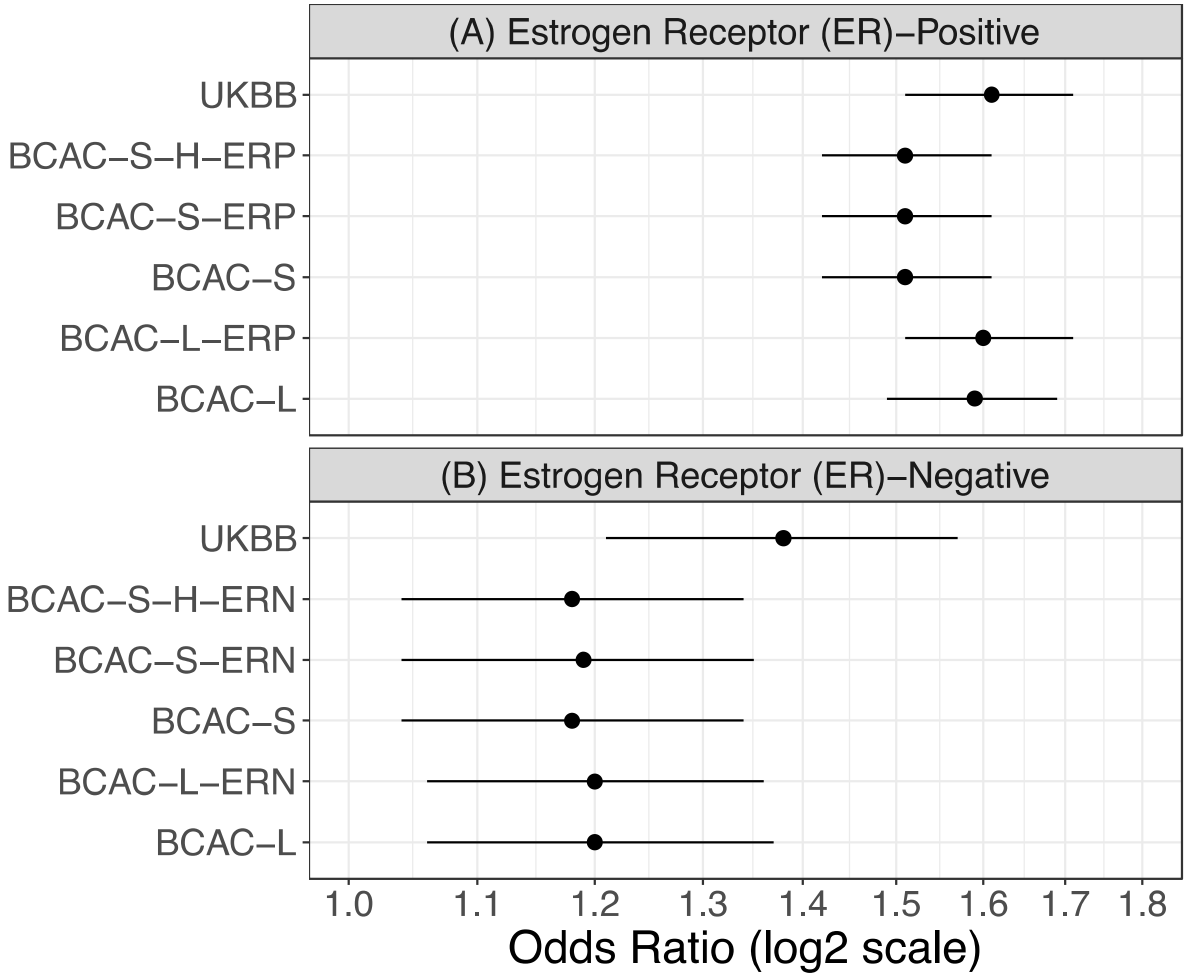

© 2021 Liu C et al. *JAMA Network Open.*

eFigure 4. The association of the different PRSs and breast cancer (BC) risk in women of European, African, and Latinx ancestries, stratified by eMERGE study sites

Odds ratios and 95% confidence intervals of BC per standard PRS unit increase in women of different ancestries and stratified by eMERGE site are shown. Analysis is not conducted for Latina and African American for some sites due to limited sample size (cases < 10). Analysis is not conducted for European ancestry in Columbia due to limited sample size (cases < 10). Marshfield Clinic=mrsh; Vanderbilt University=vand; Kaiser Permanente Washington Health Research Institute / University of Washington=kpuw; Mayo Clinic=mayo; Northwestern University=nwun; Geisinger=geis; Columbia = colu; Partners Healthcare=harv;.Mount Sini = Mtsi

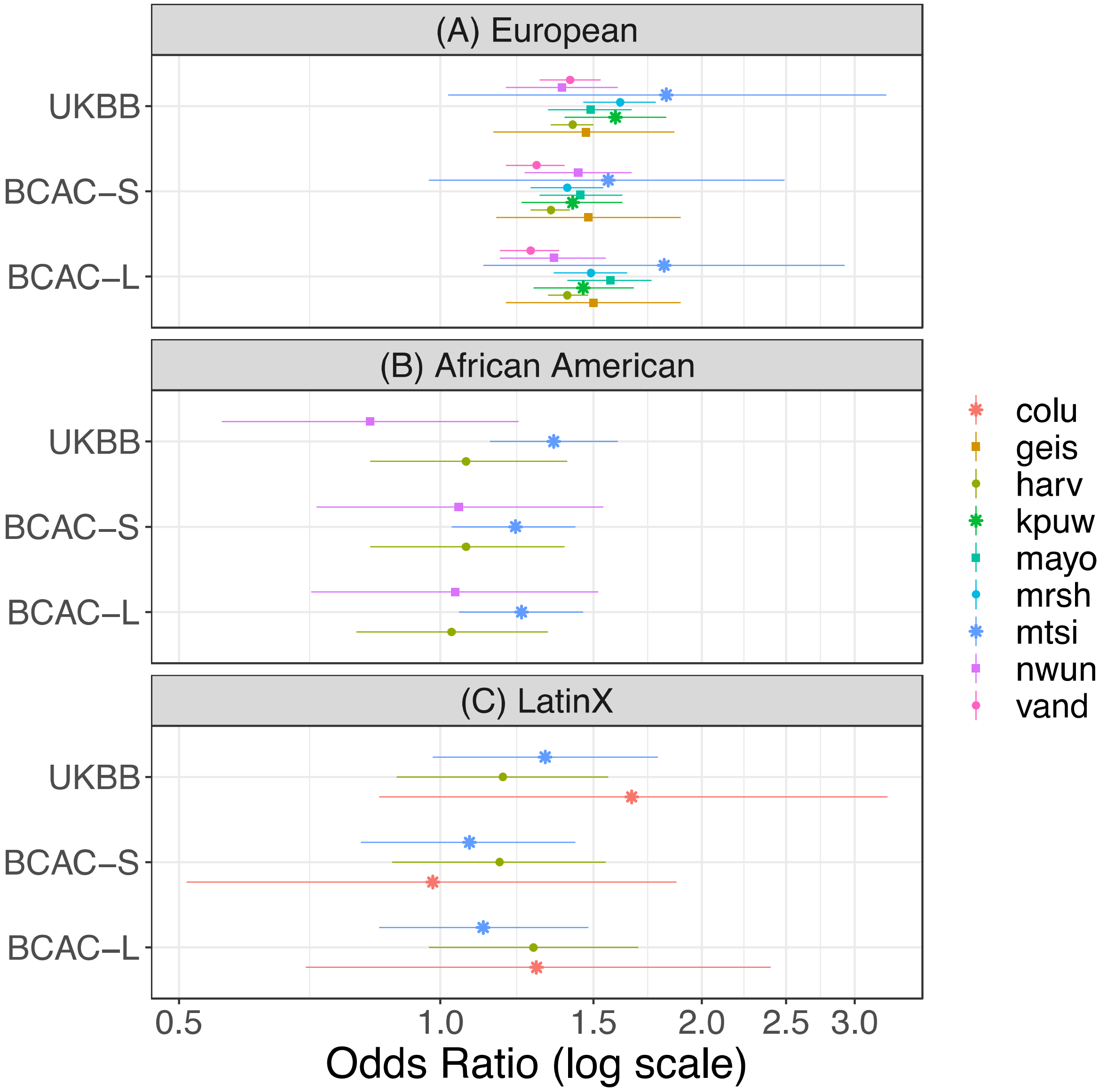

© 2021 Liu C et al. *JAMA Network Open.*

eFigure 5. The association between different PRSs generated by various filter stacks and overall breast cancer for women

Odds ratios and 95% confidence intervals are presented per standard PRS unit increase. f1(filter stack 1): no allele mismatch, no 3+allele, no 3+allele, and Genotyped or imputed in eMERGE; f2(filter stack 2): no allele mismatch, no 3+allele, no 3+allele, Genotyped or imputed in eMERGE, imputated Rsq>0.3, HWE>1e-6 in ANY ancestry, and MAF>0.005; f3(filter stack 3): no allele mismatch, no 3+allele, no 3+allele, Genotyped or imputed in eMERGE, imputated Rsq>0.8, HWE>1e-6 in ALL ancestry, and MAF>0.01;

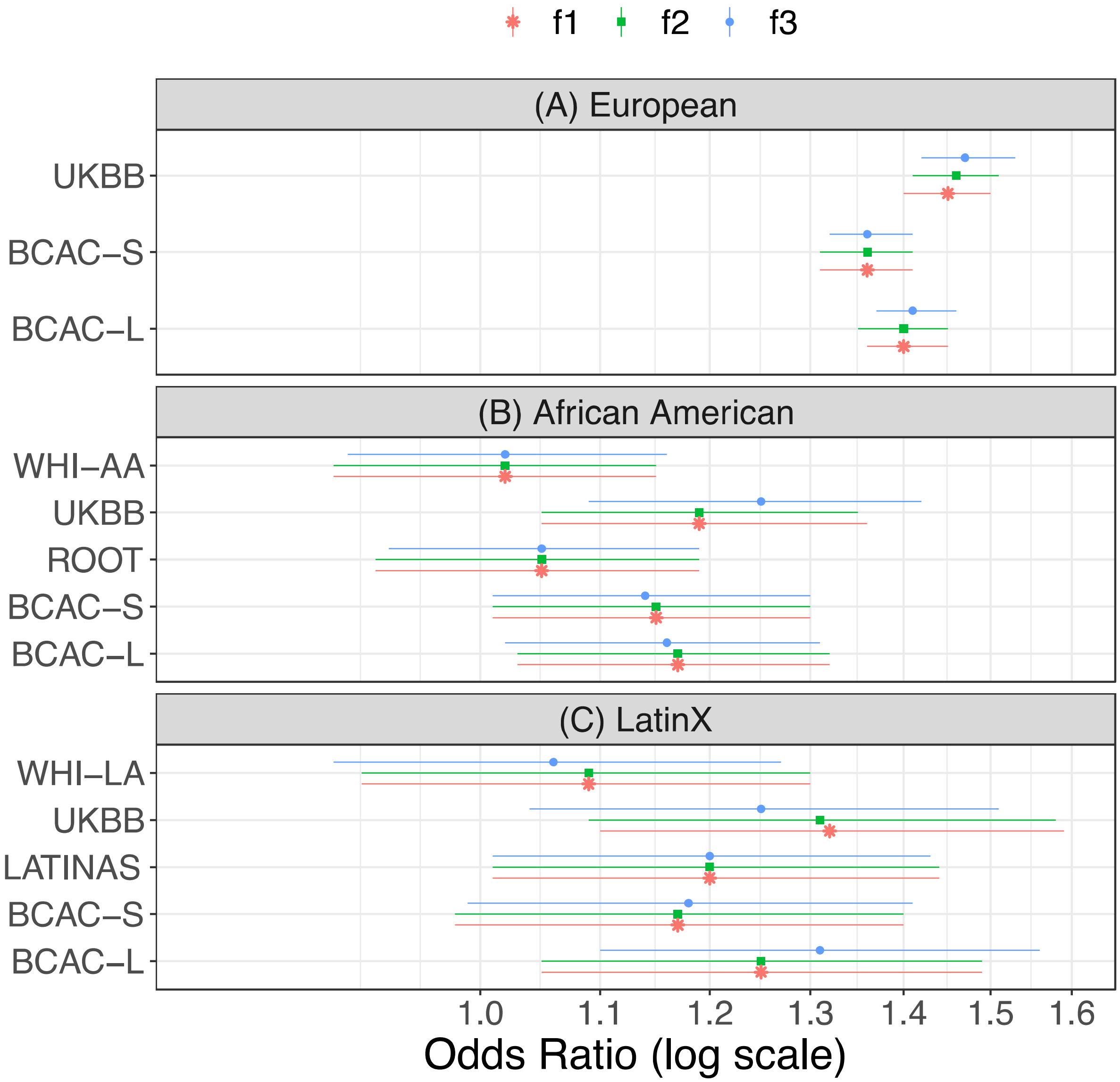

© 2021 Liu C et al. *JAMA Network Open.*

eMethods

Phenotyping

We restrict our analysis within women with age > 18. We defined a woman as a breast cancer case if there was at least one occurrence of the female breast cancer diagnostic codes (**eTable 1**) or at least two occurrences (from distinct calendar days) of breast cancer history codes (**eTable 2**) if she had no breast cancer diagnostic codes. Controls were defined having none of the female breast cancer diagnostic or history codes (**eTables 1 and eTable 2**). We further excluded participants without EMR data available. The breast cancer phenotyping algorithm has been validated by a chart review and a 95% positive predictive value for cases and negative predictive value for controls were achieved (https://phekb.org/phenotype/breast-cancer). Breast cancer cases included women with stage 0-IV breast cancer, which includes women with ductal carcinoma in-situ (stage 0). Women with stage 0 breast cancer often require definitive treatment with complete surgical resection, radiation therapy, and adjuvant hormonal therapy, and are therefore included as cases. Women with a diagnosis of benign breast disease, including those with lobular carcinoma in situ were excluded from the case definition.

For all sites e xcept for Mount Sini, we further classified breast cancer cases into subtypes by tumor ER status, based on tumor registry data or information extracted from a breast pathology report following a biopsy or surgical resection. If the breast pathology/tumor registry information was unavailable or if the ER or progesterone receptor (PR) status was missing or unknown, we queried for hormonal therapies on the medication list (**eTable 3**) after breast cancer diagnosis. We defined a breast cancer case as ER-positive if at there was at least one medication listed. If the breast pathology/tumor registry data was unavailable (or ER/PR status was missing), and no hormonal therapies were identified, then we classified the breast cancer case as subtype unknown. We also extracted breast cancer family history information from tumor registry or based on ICD9/10 codes 'V16.3' or 'Z80.3'.

PRS Models

The first two PRS models evaluated in this study (BCAC-S and BCAC-L) were developed by the Breast Cancer Association Consortium (BCAC) based on approximately 90,000 breast cancer cases and 75,000 control women of EA [1]. BCAC-S, which includes 313 variants, was developed using a hard-thresholding approach, and BCAC-L, which includes 3820 variants, was developed using a lasso regression-based approach [1]. We used information on included variants and corresponding effect sizes from the original publication (Mavaddat et al, 2019: Supplementary

© 2021 Liu C et al. *JAMA Network Open.*

Table S7 and Table S8) to construct both overall breast cancer and subtype-specific PRSs. The third PRS model we examined includes 5218 variants and was developed using the UK Biobank (UKBB) data based on summary statistics from the BCAC GWAS study and was optimized based on approximately 190,000 women in UKBB of EA (including approximately 3,000 breast cancer cases) using the Ldpred method [2]. To reconstruct this PRS, we downloaded variant and effect size information (BreastCancer_PRS_PT_r2_0.2_p_0.0005_v3.zip) from http://www.broadcvdi.org/informational/data.

We also included two PRS models developed in or adapted to LA women, LATINAS [3] and WHI-LA [4], which selected an optimized set of variants using a validation set comprised of LA women. LATINAS, which includes 179 variants, used previously published GWAS summary statistics from BCAC GWAS study in EA women and an optimized variant set based on a validation dataset consisting of approximately 4,600 LA breast cancer cases and approximately 7,600 control women [3]. We used Supplementary Table 2 (from Shieh et. al, 2019) to construct the PRS model, LATINAS. WHI-LA, comprised of 71 variants re-estimates the effect sizes of previously identified risk loci in EA based on ~3000 self-identified LA women from the Women's Health Initiative (WHI) SNP Health Association Resource (SHARe) [4]. We used information reported (the OR column) in the supplementary Table 4 (from Allman et al., 2015) on unadjusted ORs for individual variants in Latino ancestry to construct WHI-LA.

We also included two PRS models developed in AA women, WHI-AA[4] and ROOT[5]. WHI-AA includes 75 variants and is based on a validation set of ~ 4,000 AA women enrolled in the WHI (the OR column in the Supplementary Table 2 in Allman et al, 2015) [4]. Another model developed in AA women was developed using summary statistics from GWASs of breast cancer in the African diaspora (The Root consortium) which included 3686 participants of AA [5]. We used their validated PRS model consisting of 34 variants (Supplementary Table 2 in Wang et al, 2018) with consistent direction among previous studies (i.e. WHI, BCAC, and AABC), and the odds ratio source used is obtained from the WHI study based on African American populations.

To calculate breast cancer subtype related PRS, we included PRSs developed for breast cancer subtypes (ER-positive and ER-negative BCs) by Mavaddat et al.[1]. BCAC-S-ERP (for ER-positive BC) and BCAC-S-ERN (for ER-negative) were PRS models consisting of 313 variants with effect size optimized based on ER-subtypes. BCAC-S-H-ERP and BCAC-S-H-ERN were hybrid models consisting of the same 313 variants where the optimum effect size was obtained when a subset of variants in the base model were given subtype-specific weights if the breast

© 2021 Liu C et al. *JAMA Network Open.*

cancer subtype association is significant, while the remaining variants were given the effect size for overall BC. Subtype specified PRS models (BCAC-L-ERP and BCAC-L-ERN) were also constructed for the BCAC-L model, which included 5,218 variants.

PRS Calculation

To estimate each PRS, we excluded ambiguous variants (i.e. C/G and A/T), variants with allele mismatches even after strand flipping, and variants with more than 3+allele from each PRS model. We used PLINK 1.9 [6] to calculate each PRS as a weighted sum using the --score function.

Estimation of absolute risk of BC

To explore the potential clinical utility of PRS models that were significantly associated with breast cancer, individulas were group into tertiles of PRS distribution. We estimated cumulative risk of breast cancer for high PRS risk (top tertile), moderate PRS risk (middle tertile) and low risk (bottom tertile) individuals in each ancestry using iCARE [7]. We chose the PRS that was the most strongly associated with breast cancer within each ancestry (UKBB in EA and AA women, and BCAC-L model in LA) to estimate absolute risks. Ethnicity, race, and age-specific breast cancer rates in the US were from the Surveillance, Epidemiology, and End Results Program (SEER) [8]. Ethnicity, race, and age-specific mortality rates in the US were extracted from Centers for Disease Control and Prevention's WONDER online database (http://wonder.cdc.gov/ucd-icd10.html) as competing risks.

© 2021 Liu C et al. *JAMA Network Open.*

eReferences

1. Mavaddat N, Michailidou K, Dennis J, et al. Polygenic Risk Scores for Prediction of Breast Cancer and Breast Cancer Subtypes. *Am J Hum Genet.* 2019;104(1):21-34.
2. Khera AV, Chaffin M, Aragam KG, et al. Genome-wide polygenic scores for common diseases identify individuals with risk equivalent to monogenic mutations. *Nat Genet.* 2018;50(9):1219-1224.
3. Shieh Y, Fejerman L, Lott PC, et al. A Polygenic Risk Score for Breast Cancer in US Latinas and Latin American Women. *J Natl Cancer Inst.* 2020;112(6):590-598.
4. Allman R, Dite GS, Hopper JL, et al. SNPs and breast cancer risk prediction for African American and Hispanic women. *Breast Cancer Res Treat.* 2015;154(3):583-589.
5. Wang S, Qian F, Zheng Y, et al. Genetic variants demonstrating flip-flop phenomenon and breast cancer risk prediction among women of African ancestry. *Breast Cancer Res Treat.* 2018;168(3):703-712.
6. Chang CC, Chow CC, Tellier LC, Vattikuti S, Purcell SM, Lee JJ. Second-generation PLINK: rising to the challenge of larger and richer datasets. *Gigascience.* 2015;4:7.
7. Pal Choudhury P, Maas P, Wilcox A, et al. iCARE: An R package to build, validate and apply absolute risk models. *PLoS One.* 2020;15(2):e0228198.
8. Chatterjee N, Shi J, García-Closas M. Developing and evaluating polygenic risk prediction models for stratified disease prevention. *Nature Reviews Genetics.* 2016;17:392.

© 2021 Liu C et al. *JAMA Network Open.*
